# Constructing gene similarity networks using co-occurrence probabilities

**DOI:** 10.1186/s12864-023-09780-w

**Published:** 2023-11-21

**Authors:** Golrokh Mirzaei

**Affiliations:** Department of Computer Science and Engineering, The Ohio State University, Marion, USA

**Keywords:** Similarity network, Gene network, Probability, Co-ccurrence, Cancer

## Abstract

Gene similarity networks play important role in unraveling the intricate associations within diverse cancer types. Conventionally, gauging the similarity between genes has been approached through experimental methodologies involving chemical and molecular analyses, or through the lens of mathematical techniques. However, in our work, we have pioneered a distinctive mathematical framework, one rooted in the co-occurrence of attribute values and single point mutations, thereby establishing a novel approach for quantifying the dissimilarity or similarity among genes. Central to our approach is the recognition of mutations as key players in the evolutionary trajectory of cancer. Anchored in this understanding, our methodology hinges on the consideration of two categorical attributes: mutation type and nucleotide change. These attributes are pivotal, as they encapsulate the critical variations that can precipitate substantial changes in gene behavior and ultimately influence disease progression. Our study takes on the challenge of formulating similarity measures that are intrinsic to genes' categorical data. Taking into account the co-occurrence probability of attribute values within single point mutations, our innovative mathematical approach surpasses the boundaries of conventional methods. We thereby provide a robust and comprehensive means to assess gene similarity and take a significant step forward in refining the tools available for uncovering the subtle yet impactful associations within the complex realm of gene interactions in cancer.

## Introduction

Similarity measure play a crucial role in discerning the likenesses between entities within interaction networks. Within these networks, entities are symbolized as nodes, and the connections between nodes are designated as edges, determined by a particular similarity measure. Such measures find utility across various interaction networks, encompassing gene similarity networks, patient similarity networks, protein-to-protein interaction networks, social networks, and beyond.

The study of gene interaction profile similarity includes two primary approaches. First, association indices emerge as pivotal metrics that leverage statistical and mathematical modeling to quantitatively gauge the similarity between genes. Second, the study delves into epistatic interactions, which are elucidated through experimental definition and investigation. These interactions manifest in networks represented as graphs, where genes are nodes and edges depict the inter-gene similarity. The process of establishing similarity between gene profiles involves experimental specifications based on gene interactions within the genome. Consequently, network edges connect pairs of genes that interact with analogous gene sets. Notably, the choice of similarity measure employed between gene vectors wields a discernible influence over network properties and the detection of gene modules within these networks. Glazko et al. [1] undertook an examination of various distance measures between binary vectors, revealing their characteristic properties and performance across diverse genome analysis tasks. Barido-Sottani [2], meanwhile, applied four distinct similarity measures to yeast genetic interactions, yielding unique gene modules for each measure. In a distinct investigation [3], gene similarity networks were meticulously constructed based on the proteomes of eukaryotes, prokaryotes, viruses, and plasmids.

Numerous techniques have emerged, primarily within patient similarity networks, aimed at uncovering the similarities inherent in patients' profiles. These networks are constructed through the aggregation of diverse patient data (features), culminating in a unified framework that embodies the essence of patient similarity. Each node within these networks symbolizes an individual patient, while the edges are emblematic of the pairwise likeness between patients with regard to a specific feature. One of the widely adopted techniques is Similarity Network Fusion (SNF), an innovative technique in genomic data integration introduced by Wang et al. [4]. SNF pioneers the construction of patient similarity networks for individual data types, subsequently iteratively integrating them until convergence is achieved, resulting in a consolidated, fused network. Delving into advanced methodologies, Navaz et al. [5] propounded a deep learning-centric approach that leverages convolutional neural networks for the construction of patient similarity networks. In a complementary perspective, Gliozzo et al. [6] offered insights into a range of integration methods for patient similarity networks.

The applications of patient similarity networks extend to patient clustering [7, 8], wherein patients sharing similar characteristics are grouped together. Additionally, techniques abound for the prioritization of disease genes through protein–protein interaction (PPI) networks. Within these networks, proteins are nodes, and the edges signify interactions between them. Notably, Tian et al. [9] harnessed SNF [4] to craft an integrated gene similarity network from individual gene (protein) similarity networks, enabling the identification of disease genes. Kovacs et al. [10], on the other hand, introduced a node (protein) similarity metric grounded in shared neighbors, emphasizing that proteins with common neighbors exhibit akin interaction interfaces. Through these multifaceted methodologies, the exploration of patient or gene similarity and the identification of disease-related genes acquire new dimensions of depth and insight.

The exploration of molecular modules within genetic networks is notably influenced by the approach used to measure the similarity between profiles of gene interactions within a cell. Given the absence of a definitive method for selecting the *optimal* measure, it is advisable to embrace various measures with distinct mathematical properties, which may identify different sets of connections between genes [2]. Over the past decade, statistical and mathematical techniques aimed at identifying associations between genes have garnered significant attention. Bass et al. [11] offer a comprehensive overview of frequently employed association indices, encompassing noteworthy measures like the Jaccard index and the Pearson correlation coefficient. Further, the performance of various association indices across a spectrum of biological network analyses is described in their work. This comprehensive investigation outlines how different indices fare in diverse types of analyses within biological networks.

Genetic association analysis using somatic mutations is an effective methodology to understand the functional impact of somatic mutations and to reveal the potential impact of somatic mutations on molecular or clinical features [12]. Mutation plays an important role in cancer evolution as it alters the gene function in ways that may lead to cellular transformation and immortalization. The most common type of DNA mutation is the single alteration of a nucleotide by substitution of another one which is known as point mutation [13]. Point mutations can lead to twelve distinct types of base substitutions, involving the four bases: T, C, G, and A. In this study, we focus on two categorical attributes of mutations: mutation types (such as nonstop, silent, etc.) and nucleotide transformations, described as follows.

Mutations are categorized into various types based on the impact they exert on the sequence of the translated protein. A mutation that results in the substitution of one amino acid with another within a protein's sequence is termed a missense mutation, also referred to as non-synonymous mutation. In contrast, if a mutation in the DNA sequence does not lead to any alteration in the amino acid sequence, it is termed a synonymous mutation or silent mutation. As an illustrative example, consider a C $\to$ T mutation. This alteration would cause the codon CTT, which normally codes for the amino acid leucine, to be changed to ATT, leading to the encoding of isoleucine instead [13]. This type of mutation can have diverse effects on the protein's structure and function, ranging from negligible impact to significant functional alterations, depending on the specific amino acids involved and their roles within the protein's three-dimensional structure and interactions. The nonsynonymous to synonymous ratio is significantly lower in cancer-related genes compared to that in other genes. This suggests that most nonsynonymous substitutions drastically affect the function of a gene [14, 15].

A point mutation can also change a codon for an amino acid into one of the terminations, or STOP codons, namely TAG, TAA, and TGA. Terminating mutations, also known as nonsense mutations, cause the truncation of the open reading frame. Nonstop or stop-loss mutations convert a stop into a sense codon, resulting in translation into the 3′ untranslated region as a nonstop extension mutation to the next in-frame stop codon or as a readthrough mutation into the poly-A tail [16]. A genetic alteration in the DNA sequence that occurs in the specific site at which splicing takes place during the processing of precursor messenger RNA into mature messenger RNA, is called splice-site mutation. The splice site is the boundary of an exon and an intron, in which mutation can disrupt RNA splicing resulting in the loss of exons or the inclusion of introns and an altered protein-coding sequence.

The nucleotide transformation or nucleotide change refers to a substitution, deletion, or insertion of a single nucleotide (A, T, C, or G) within a DNA or RNA sequence. Nucleotides are the building blocks of DNA and RNA, and the sequence of these nucleotides encodes genetic information. A change in a single nucleotide can lead to alterations in the genetic code, potentially resulting in changes to the corresponding protein or RNA molecule. For example, transformation from Adenine (A) to Thymine (T). If a DNA sequence originally contained the nucleotide “A” at a specific position and it undergoes a mutation, resulting in the nucleotide at that position being changed to “T”, then it's a transition mutation from adenine (A) to thymine (T).

The degree of similarity between two entities, particularly genes in this context, inherently hinges on the proximity of their attribute values. While gauging this proximity is straightforward for numeric attributes, it becomes challenging to capture this notion for categorical attributes. Unlike numeric attributes, categorical attributes encompass features that manifest across varying levels or categories, rather than being quantifiable numbers. This challenge becomes particularly prominent in gene similarity networks grounded in mutations, where categorical attributes are prevalent. Consider, for instance, mutation types such as missense, nonsense, and splice site. These attributes cannot be directly compared in the manner that we contrast numerical values. This encapsulates a significant hurdle encountered in the construction of gene similarity networks that rely on categorical attributes. The task of calculating similarity between categorical entities poses a critical data mining problem, particularly within the domain of unsupervised learning.

A prevalent limitation in many existing distance measures is their failure to consider the distribution of values within a dataset when assessing similarity between categorical attribute values. Unlike numeric attributes where calculating distance takes into account the inherent values, this natural consideration is not extended to categorical attributes. In unsupervised learning domain, numerous distance measures have been proposed, aiming to quantify similarity between categorical objects. Some notable examples include the Hamming distance, Jaccard coefficient, and Sokal-Michner (M-coefficient) similarity measure [17]. Ichina and Yaguchi [18] assert that the distance between two categorical values remains a constant, regardless of the specific categorical values being compared. However, this approach's effectiveness as a measure of distance is questionable, as it disregards the inherent disparities among categorical values. In recognition of these limitations, Sulc and Rezankova [19] made strides by providing a comparison of similarity measures specific to categorical data, particularly tailored to hierarchical clustering. Moreover, they introduced novel similarity measures rooted in variable entropy and variable mutability, effectively adapting the measures to the categorical data domain.

In this paper, we introduce a novel similarity measure tailored for gene association based on mutation. Our method is an extension of the co-occurrence similarity measure initially proposed by Ahmed and Dey [20]. Building upon their foundational work, we have expanded their framework to accommodate gene associations, capitalizing on two distinct attributes intrinsic to gene mutations: mutation type (such as missense or nonsense mutations) and nucleotide change (for instance, A > C or C > T alterations). To validate the efficacy of our approach, we applied it to construct gene similarity networks in the context of ovarian cancer. Through this process, we have created association networks that illuminate the complex interplay of genes in the context of mutation-driven associations. Moreover, we extended our methodology to a case study involving lung cancer, thereby reinforcing its applicability and potential.

It's noteworthy that the versatility of our approach allows for seamless extensions to other cancer types, as well as the inclusion of additional attributes, all without altering the core algorithm. By offering a robust framework for comprehending gene interactions, our work not only advances our understanding of cancer genetics but also paves the way for broader applications in the study of diverse biological systems.

## Materials and methods

### Methodology

The proposed gene similarity measure hinges on the concept of co-occurrence of attributes, as outlined in [20]. This approach is rooted in the understanding that the similarity between two attribute values is intricately tied to their interactions with other attributes. Notably, these attributes can encompass both numerical and categorical data.

Qualitative variables, denoted as $Y$, is called categorical if their range, represented as $\gamma$, lacks any inherent internal structure. Consequently, when dealing with two categories $x$ and $y$ within $Y$ ($x,y \in Y )$, a distinction can be made between $x=y$ (indicating equality) and $x\ne y$ (indicating inequality). It is important to acknowledge that quantifying the similarity between categorical data proves challenging using existing measures primarily designed for numerical data, such as Jaccard or Hamming distance.

Our methodology involves computing the distance between gene attributes of the same type with respect to other attributes. This property is fundamental in the calculation of gene similarity measures, as it takes into consideration the intricate relationships between attributes. Table 1 illustrates the two specific attributes we utilize for the computation of gene similarity measures. This comprehensive approach enriches our understanding of the multifaceted nature of gene interactions and the role attributes play in defining their relationships.

**Table 1:** Attributes and corresponding categorical values used in gene similarity measure

**Mutation type**	Missense	Nonsense	RNA	Silent	Splice_Site	Nonstop						
Nucleotide change	C > A	T > A	A > G	C > T	G > T	G > A	T > G	C > G	A > C	T > C	G > C	A > T

### Similarity between attributes

Let’s consider two values $x,y\in {A}_{i}$, where ${A}_{i}$ is $i$ ^th^ categorical attribute. The distance between $x$ and $y$ are computed by taking into account the overall distribution of these two values across the dataset along with their co-occurrence with values from other attributes. In our analysis, we consider two categorical attributes denoted as ${A}_{i}$ and ${A}_{j}$. The similarity $\delta (x,y, {A}_{j})$ between pair $(x,y)$ with respect to $j$ ^th^ attribute is defined as:

1 $$\delta \left(x,y, {A}_{j}\right)=\mathrm{ max }\left({P}_{i}^{x} \left(w\right)+ {P}_{i}^{y} \left(\sim w\right)\right)-1$$

where $w$ represents a subset of ${A}_{j}$ ($j$ ^th^ attribute) values over the mutation set,$\left(\sim w\right)$ is the complementary set of values occurring for attribute ${A}_{j}$, and ${P}_{i}^{x}(w)$ is the conditional probability that the output class is one of the classes of $w$ given that $i$ ^th^ attribute has the value$x$. Given a set with cardinality$m$, the number of possible subsets generated from the set is${2}^{m}$, thus there are ${2}^{\left|{A}_{i}\right|}$ value possible for$w$.

Let for subset $w$, ${P}_{i}^{x} \left(w\right)+ {P}_{i}^{y} \left(\sim w\right)-1$ has maximum value and assuming that $\omega$ is subset of classes, $\omega \subset w$, that maximize the value of Eq. 1. If $(x,\omega )$ and $(y,\omega )$ are similarly connected, then $x$ and $y$ are similar to each other and value of ${P}_{i}^{x} \left(w\right)+ {P}_{i}^{y} \left(\sim w\right)-1$ will be small. We can say that similarity between $x$ and $y$ with respect to ${A}_{j}$ depends upon co-occurrence probabilities of $(x,\omega )$ and $(y,\omega )$ [20].

The similarity/dissimilarity between two categorical values are computed with respect to every other attribute of dataset. The average value of distances will give the distance $\delta (x,y)$ ($x,y$ belong to $i$ ^th^ attribute) between two categorical values in that dataset. A substantial distance between two attribute values suggests their significance within the dataset. Furthermore, this observation implies a high probability that data objects possessing these attribute values belong to distinct clusters. The similarity measure is computed as:

2 $$\delta \left(x,y\right)= \frac{1}{m-1} \sum_{j=1,\dots m,i\ne j}{\delta }^{i,j}\left(x,y\right)$$

where ${\delta }^{i,j} \left(x,y\right)$ is the similarity measure between pair $(x,y)$ in regard to both attributes $i$, $j$, and $m$ is total number of attributes.

Consider the definitions, these properties will be upheld:

1. $$0\le \delta \left(x,y\right)\le 1$$  
2. $$\delta \left(x,y\right)=\delta \left(y,x\right)$$
3. $$\delta \left(x,x\right)=0$$

The algorithm for calculating the distance between every pair of attribute values across all attributes is detailed in Algorithm1.

We consider two attributes for gene similarity network (mutation type, nucleotide change). Let us consider the example of mutations dataset in Table 1. Procedure to compute the similarity between the two genes FAM171B and ABCA6, are shown in Tables 2 and 3.

**Algorithm 1.**
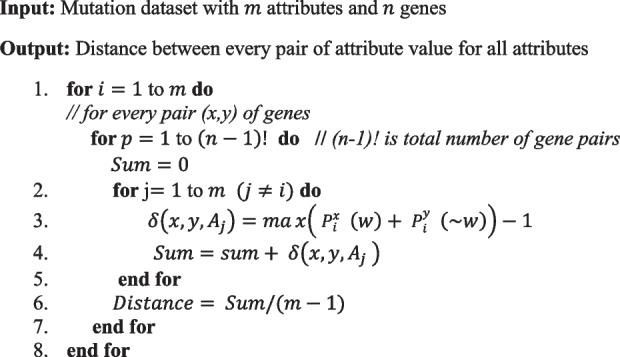
Gene Distance/Similarity

**Table 2:** An example of mutation dataset

Gene	Mutation type	Nucleotide change
FAM171B	Missense	AA → AC
FAM171B	Nonsense	GG → GT
FAM171B	Missense	GG → GA
ABCA6	Missense	GG → GT

**Table 3:**
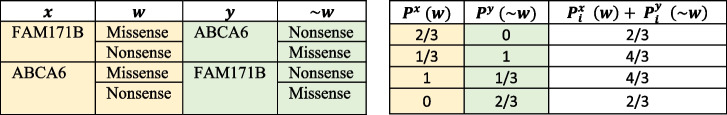
Computation of $\delta \left(FAM171B, ABCA6\right)$ with respect to mutation type

According to Tables 2 and 3, and Eq. 1, the similarity in respect to mutation type is as:

$${\delta }_{1}\left(\mathit{FAM}171B, \mathit{ABCA}6\right)=\mathrm{max}\left({P}_{i}^{x} \left(w\right)+ {P}_{i}^{y} \left(\sim w\right)\right)-1=\frac{4}{3}-1= \frac{1}{3}$$

This formula is calculated based on the following procedure:

$${\delta }_{11}\left(\mathit{FAM}171B, \mathit{ABCA}6\right)={P}_{1}\left(missense\right|FAM171B)+ {P}_{1}(nonsense|ABCA6) = \frac{2}{3}+0=\frac{2}{3}$$

$${\delta }_{12}\left(\mathit{FAM}171B, \mathit{ABCA}6\right)={P}_{1}\left(missense\right|ABCA6)+ {P}_{1}(nonsense|FAM171B) =1+ \frac{1}{3}=\frac{4}{3}$$

$${\delta }_{1}\left(FAM171B, ABCA6\right)=\mathrm{max}({\delta }_{11}\left(FAM171B, ABCA6\right), {\delta }_{12}\left(FAM171B, ABCA6\right))-1$$

$$=\mathrm{max}(\frac{2}{3} , \frac{4}{3})-1= \frac{4}{3}-1=\frac{1}{3}$$

Similarly, according to Tables 2 and 4, and Eq. 1, the similarity in respect to nucleotide change is as:

**Table 4:**
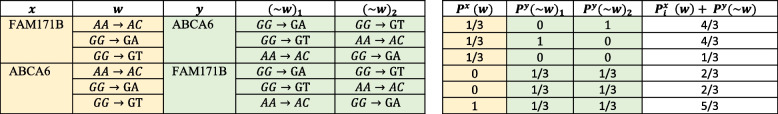
Computation of $\delta \left(FAM171B, ABCA6\right) w$ith respect to nucleotide change

$${\delta }_{2}\left(FAM171B, ABCA6\right)=\mathrm{max}\left({P}_{i}^{x} \left(w\right)+ {P}_{i}^{y} \left(\sim w\right)\right)-1=\frac{5}{3}-1= \frac{2}{3}$$

Finally, the overall similarity/distance between the two genes are computed based on Eq. 2, as follows:

$$\delta \left(FAM171B, ABCA6\right)=\frac{1}{2} \left( {\delta }_{1}\left(FAM171B, ABCA6\right)+ {\delta }_{2}\left(FAM171B, ABCA6\right)\right)=\frac{1}{2}$$

## Results and discussion

The proposed similarity network based on co-occurrence probability is performed on the ovarian cancer dataset. The dataset is obtained from TCGA (The Cancer Genome Atlas) [21] (https://www.cancer.gov/tcga) using TCGA Assembler R package [15]. The dataset contains 4638 genes and 6231 samples. The computation of the similarity measure and subsequent construction of the network are conducted within the Python 3 programming environment. For visual representation, the graphs are generated using Gephi 9 [22], a popular graph visualization tool. We derived the similarity network matrix with dimensions $4638\times 4638$, wherein each element ${s}_{i,j}$ $(0<i,j<4638)$ corresponds to the similarity between genes $i$ and$j$. The computed similarity measure is confined to a range between 0 and 1, where 0 denotes similarity with$\delta (x,y)=1$, and 1 signifies similarity with$\delta (x,y)=0$. This setup reflects that genes exhibiting a similarity value of 0 are the closest, and as the genetic distance between genes increases, the associated probability approaches 1. Given the substantial dataset volume involving 4638 genes, a graphical representation depicting all gene associations is impractical. Instead, we focused on constructing local networks of genes, with particular emphasis on genes recognized as drivers in ovarian cancer.

Several genetic factors, such as BRCA1, BRCA2, P53 (TP53), KRAS, PIK3CA, CTNNB1, and PTEN, have been correlated with ovarian cancer in genetics studies [23]. These genes assume a pivotal role in enhancing our comprehension of the genetic bedrock of ovarian cancer, thereby meriting focused attention. Given their paramount importance, our study places focused emphasis on three of these genetic factors (BRCA1, BRCA2, and KRAS). In addition to our focused investigation of three driver genes, we also extend our scrutiny to non-driver genes (AIFM1, LRRC30). Recognizing that both driver and non-driver genes may harbor concealed properties that contribute to gene interactions, this inclusive approach enriches our analysis. By examining the interplay of both types of genes, we aim to uncover hidden relationships and interactions that could wield significant influence on the complex landscape of gene interactions.

**Fig. 1:**
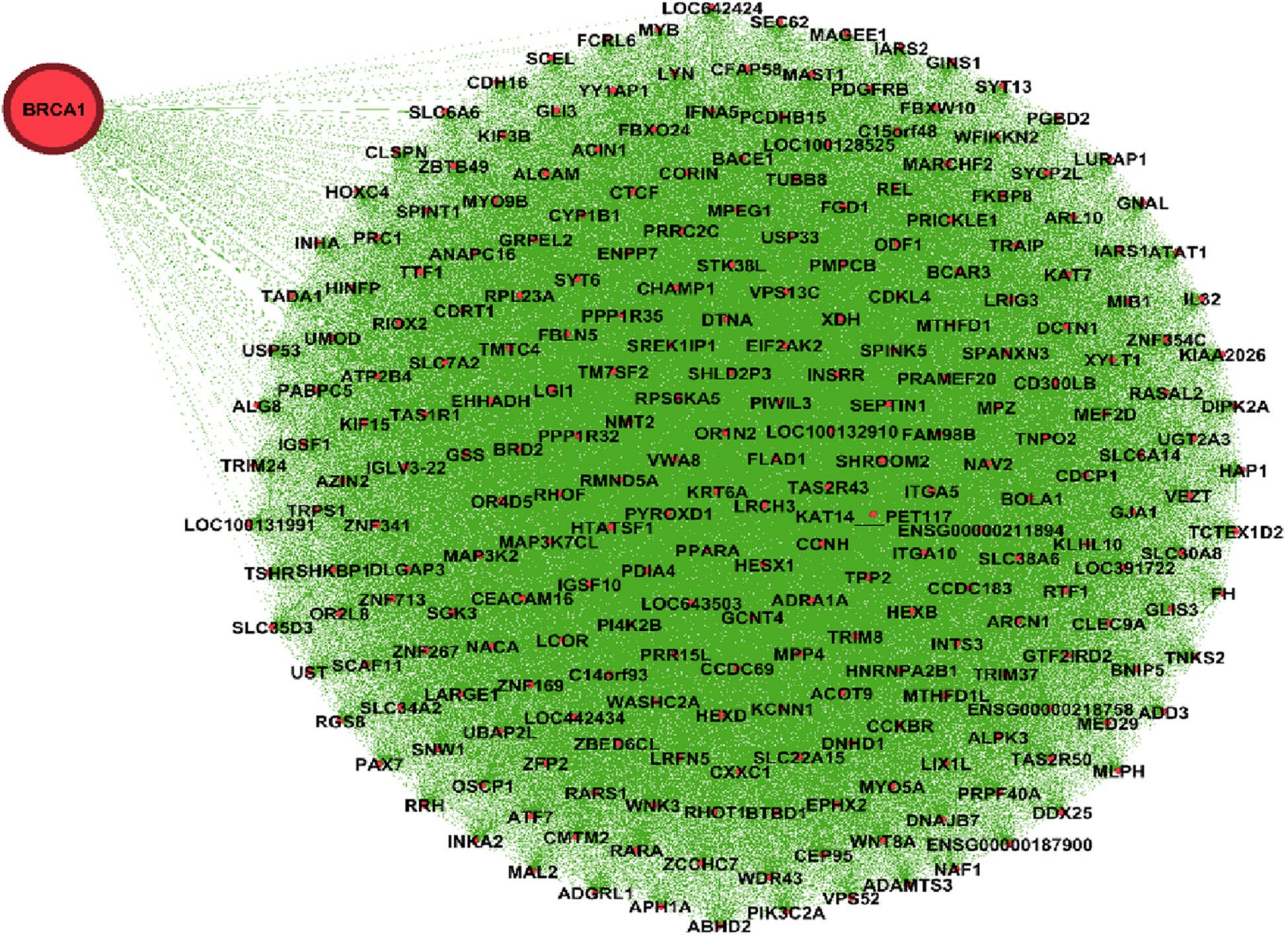
Gene similarity network for BRCA1. All genes in this network have similarity with $\delta \left(x,y\right)=1$ which indicates the *very similar* genes to BRCA1 in terms of mutation type and nucleotide change in Ovarian cancer based on co-occurrence mutation

**Fig. 2:**
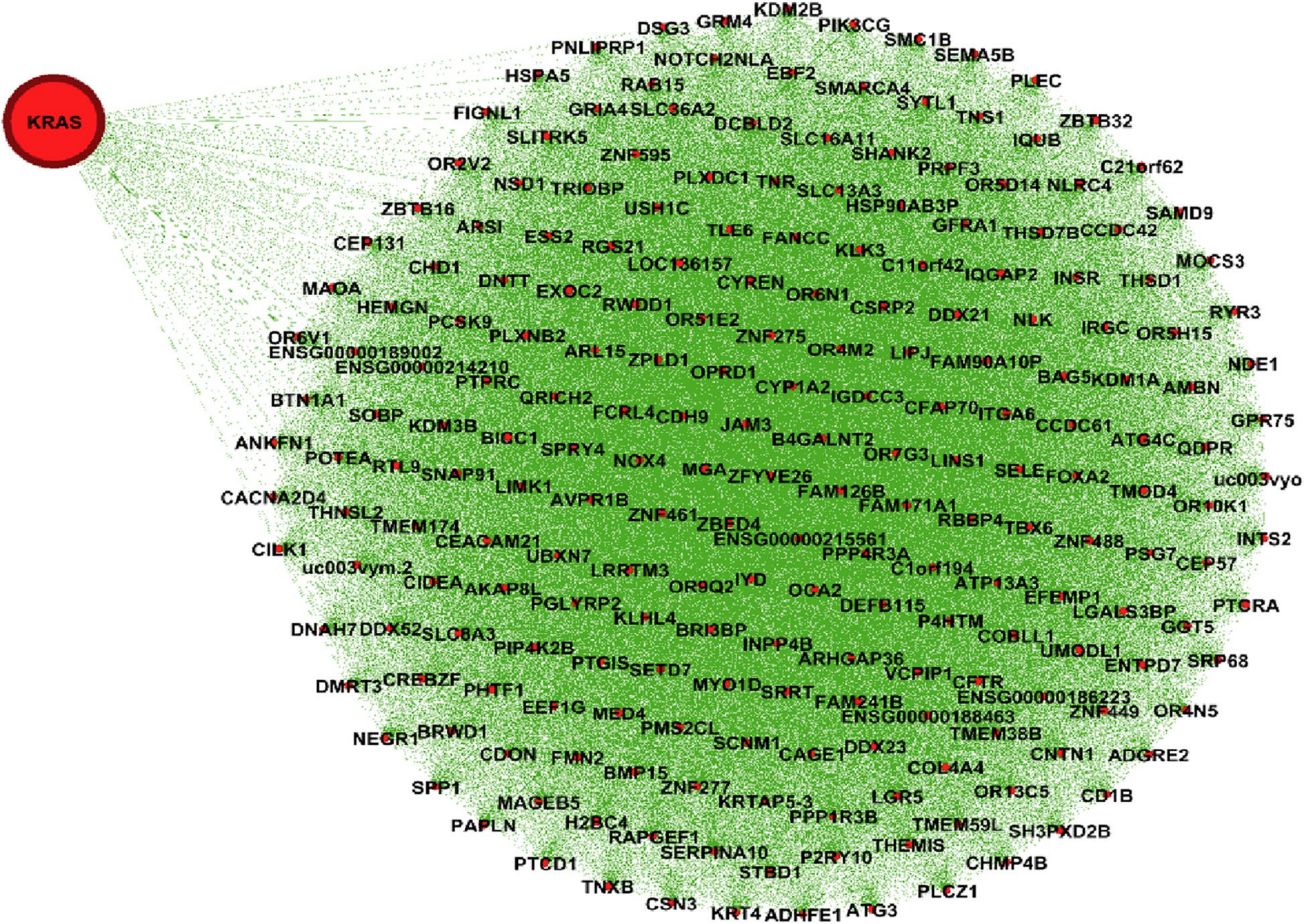
Gene similarity network for KRAS. All genes in this network have similarity with $\delta \left(x,y\right)=1$ which indicates the *very similar* genes to KRAS in terms of mutation type and nucleotide change in ovarian cancer based on co-occurrence mutation

**Fig. 3:**
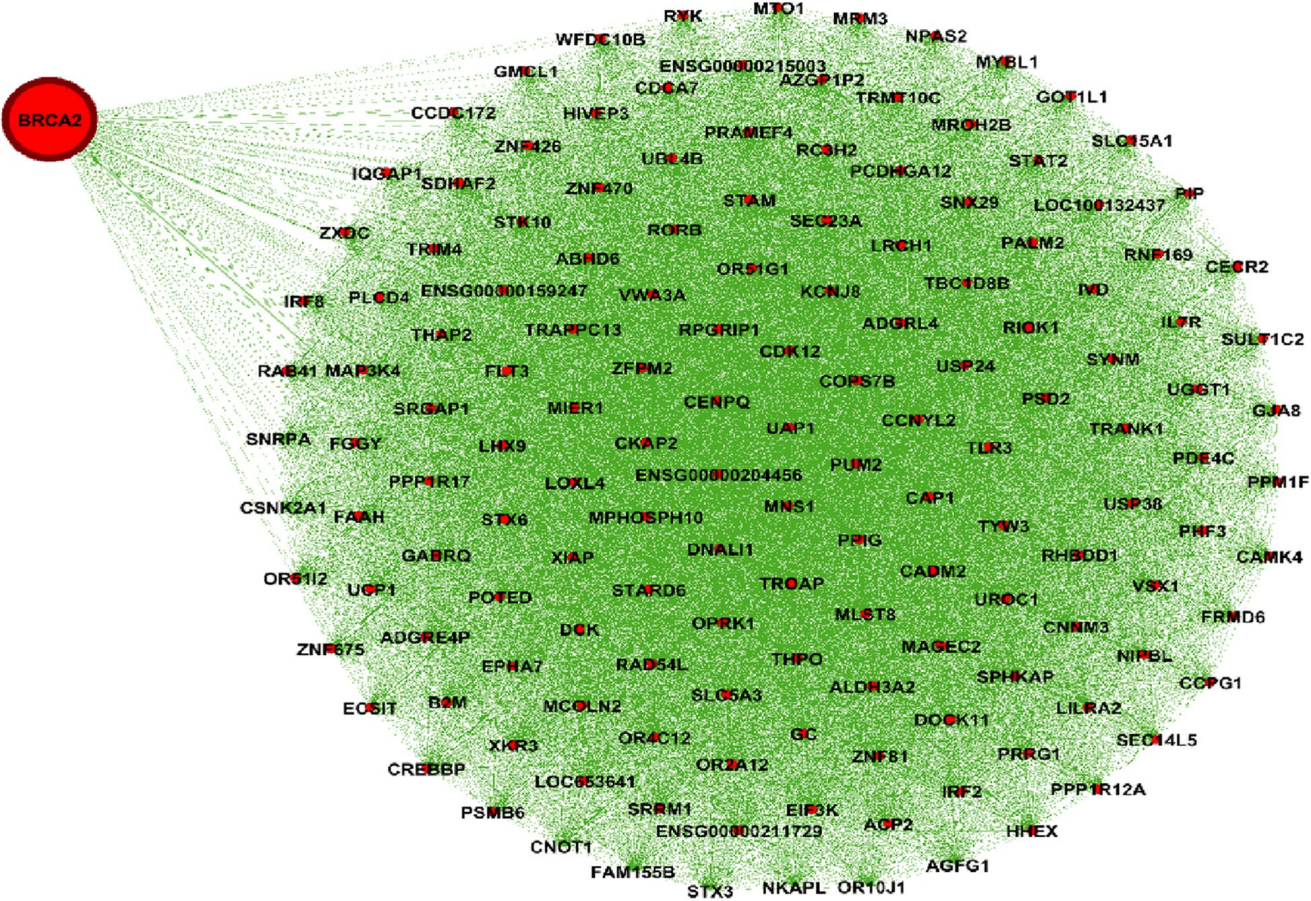
Gene similarity network for BRCA2. All genes in this network have similarity with $\delta \left(x,y\right)=1$ which indicates the *very similar* genes to BRCA2 in terms of mutation type and nucleotide change in ovarian cancer based on co-occurrence mutation

**Fig. 4:**
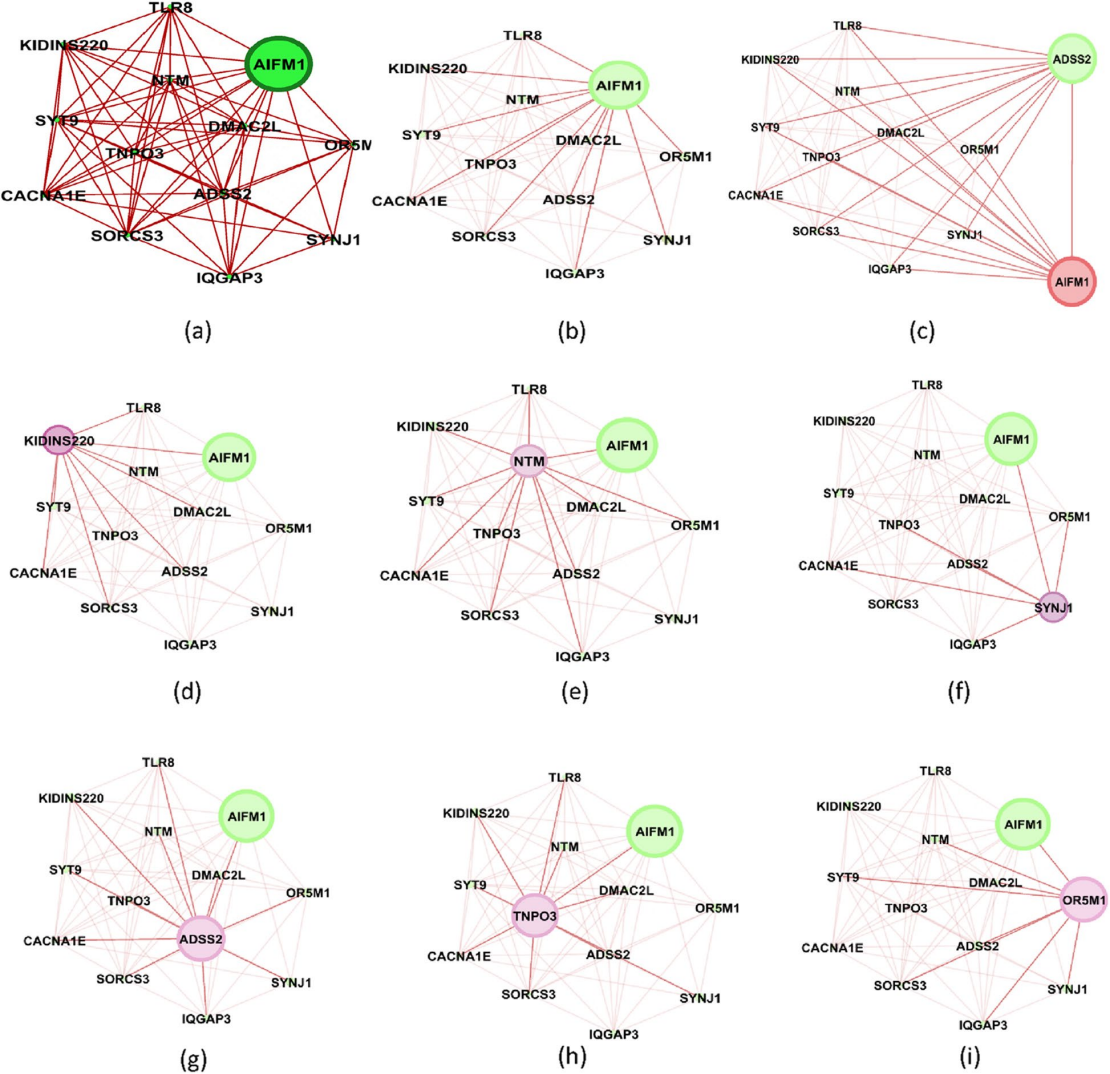
Gene similarity network of AIFM1. The similarity measure is based on co-occurrence mutation based on nucleotide change and mutation type. (**a**) similarity with $\delta (x,y)>0$ (**b**) similarity with $\delta (x,y)=1$ (**c**) The gene similarity network for AIFM1 with respect to ADSS2. All genes in this network are similar to both AIFM1 and ADSS2 with $\delta (x,y)=1$ (**d**) The gene similarity network for AIFM1 with respect to KIDDINS220. All genes in this network are similar to both AIFM1 and KIDDINS220 with $\delta (x,y)=1$ (**e**) The gene similarity network for AIFM1 with respect to NTM. All genes in this network are similar to both AIFM1 and NTM with $\delta (x,y)=1$ (**f**) The gene similarity network for AIFM1 with respect to SYNJ1. All genes in this network are similar to both AIFM1 and SYNJ1 with $\delta (x,y)=1$ (**g**) The gene similarity network for AIFM1 with respect to ADSS2. All genes in this network are similar to both AIFM1 and ADSS2 with $\delta (x,y)=1$ (**h**) The gene similarity network for AIFM1 with respect to TNPO3. All genes in this network are similar to both AIFM1 and TNPO3 with $\delta (x,y)=1$ (**i**) The gene similarity network for AIFM1 with respect to OR5M1. All genes in this network are similar to both AIFM1 and OR5M1with $\delta (x,y)=1$

**Fig. 5:**
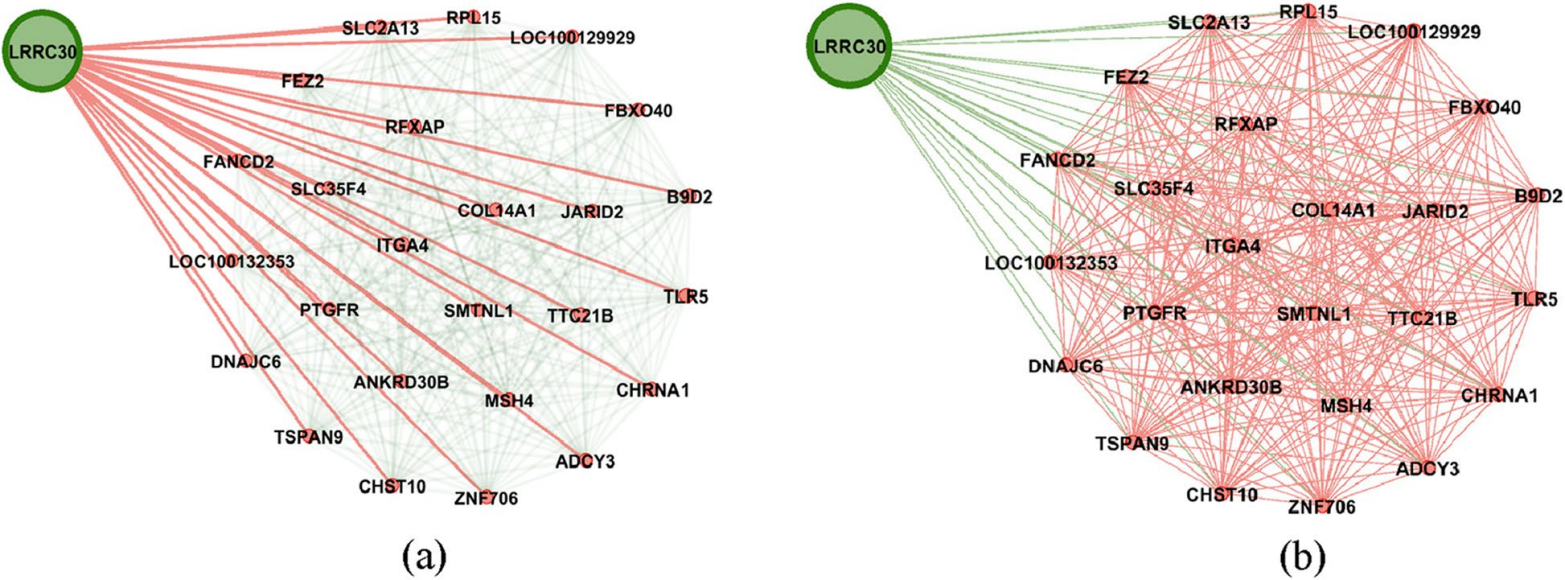
Gene similarity network for LRRC30. In (**a**), the network emphasizes gene similarity with LRRC30, while in (**b**) the network highlights the similarity among all the genes within the network

**Fig. 6:**
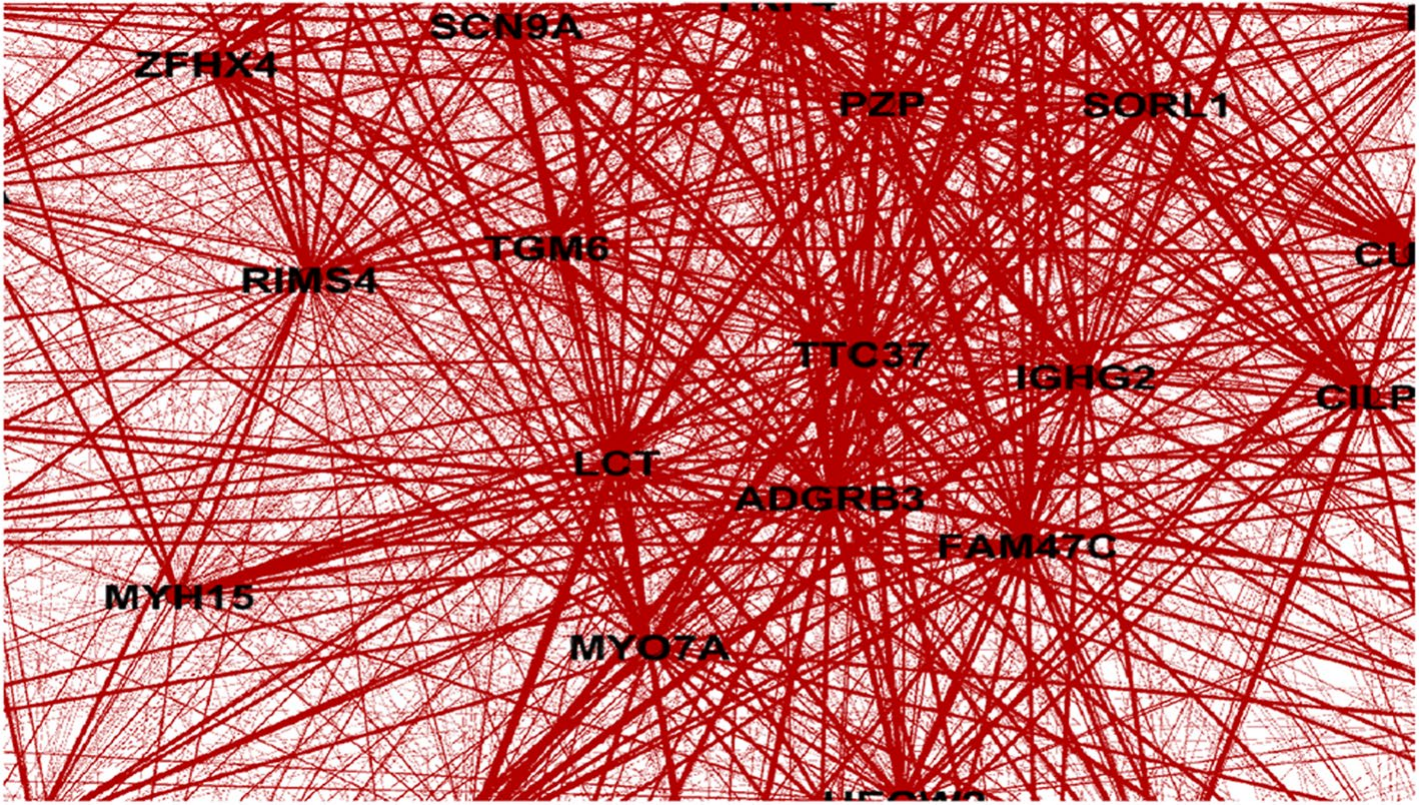
Zoom-in view of Gene similarity network

Figure 1 shows the genes’ associations within the local network of BRCA1. All genes present in this network exhibit a similarity measure 0 ($\delta (x,y)=1)$ with BRCA1, indicating a high degree of similarity in terms of both nucleotide change and mutation type, so called *very similar*. In fact, all genes within this network (such as BRCA1, KAT7, ALG8, etc.) possess one mutation characterized as missense (GG > GC). Conversely, genes absent from this network display no discernible similarity with BRCA1. For instance, ACAN is absent from this network and it is marked by a silent mutation (AA > AG), while BRCA1's mutation is classified as missense (GG > GC). Similarly, Figs. 2 and 3 show the association within local networks of KRAS and BRCA2, respectively. The trends observed in these networks mirror the insights derived from the BRCA1 network. Figure 4 provides an insight into gene associations in the local network of AIFM1, a gene not deemed a driver in ovarian cancers. Notably, this figure also highlights the specific associations between distinct genes and AIFM1. Similarly, Fig. 5 lays out the local network of LRRC30, with a detailed zoom-in view shown in Fig. 6.

**Fig. 7:**
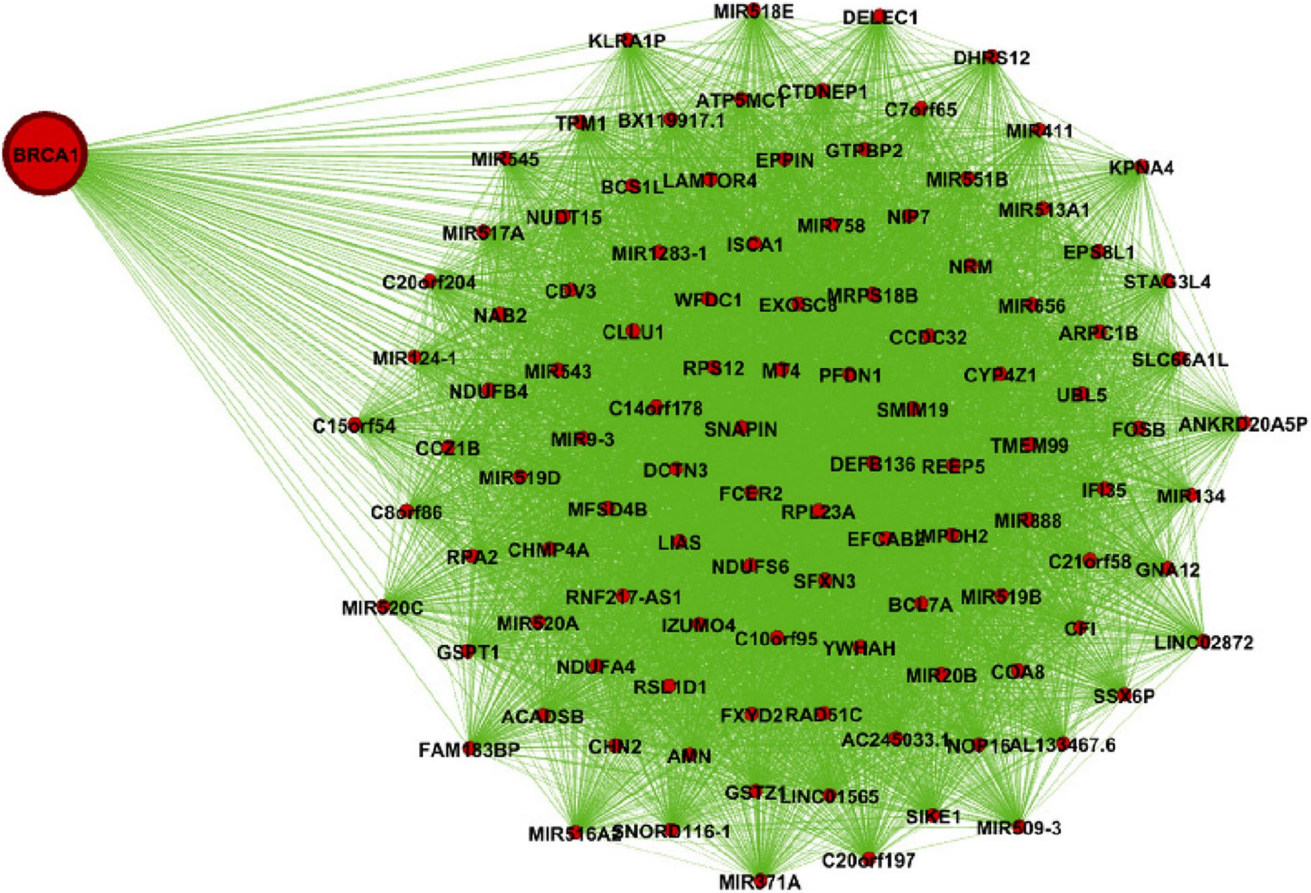
Gene similarity network for BRCA1. All genes in this network have similarity with $\delta \left(x,y\right)=1$ which indicates the *very similar* genes to BRCA1 in terms of mutation type and nucleotide change in lung cancer (LUAD) based on co-occurrence mutation

**Fig. 8:**
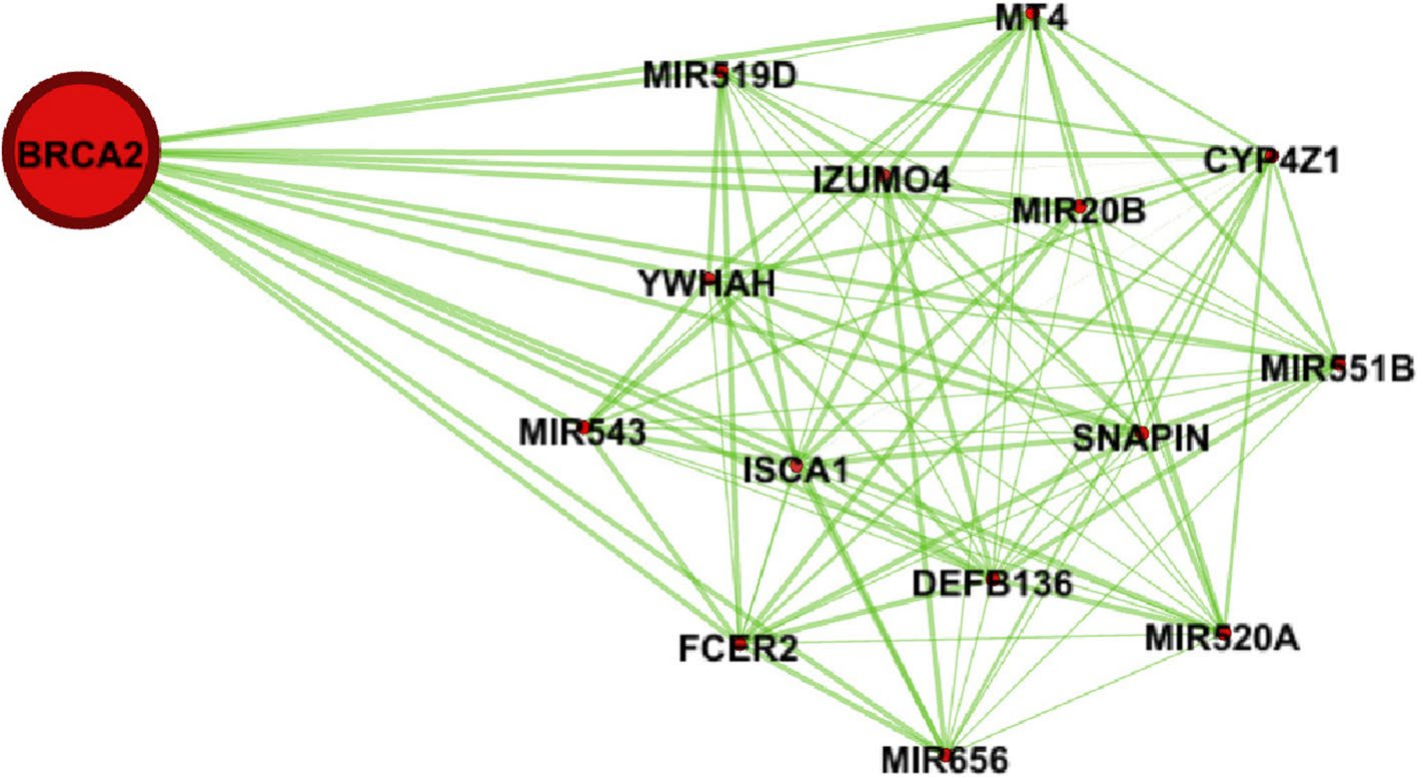
Gene similarity network for BRCA2. All genes in this network have similarity with $\delta \left(x,y\right)=1$ which indicates the *very similar* genes to BRCA2 in terms of mutation type and nucleotide change in lung cancer (LUAD) based on co-occurrence mutation

**Fig. 9:**
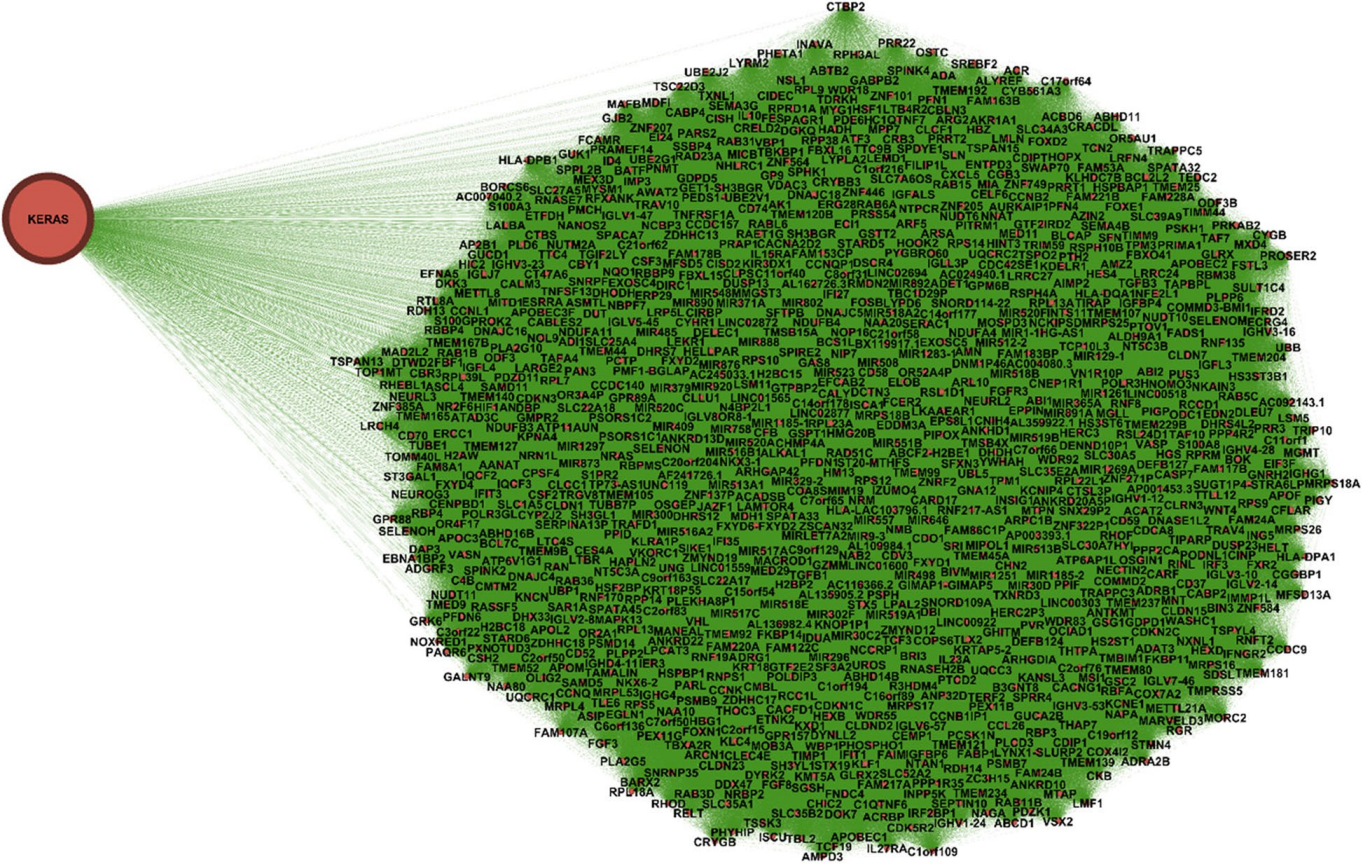
Gene similarity network for KRAS. All genes in this network have similarity with $\delta \left(x,y\right)=1$ which indicates the *very similar* genes to KRAS in terms of mutation type and nucleotide change in lung cancer (LUAD) based on co-occurrence mutation

In order to further validate our findings, we extended our analysis to another case study involving LUAD (Lung adenocarcinoma). Our investigation encompassed the examination of co-occurrence similarity networks for both driver and non-driver genes within the context of this subtype. Specifically, we focused on three genes: BRCA1, BRCA2, and KRAS where KRAS established as a driver gene, and BRCA1, BRCA2 are identified as non-driver genes in LUAD. By conducting this analysis on both driver and non-driver genes, we aimed to unravel the intricate relationships that contribute to the complex molecular landscape of LUAD. To provide a clearer visual representation, we present Figs. 7, 8, and 9, which illustrate the respective local similarity networks for BRCA1, BRCA2, and KRAS, respectively. These figures highlight the intricate connections and correlations these genes form with others, both within their own driver/non-driver category and across categories. The visualization of these networks not only deepens our understanding of gene interactions but also aids in identifying potential candidates for further exploration as therapeutic targets or prognostic markers. One interesting observation is driver gene KRAS is linked to a greater number of genes, in contrast to non-driver genes (BRCA1 and BRCA2), which exhibit fewer associations in their respective networks.

## Conclusion

In this work, we have introduced a novel mathematical model designed to quantify the likeness between pairs of genes by harnessing the co-occurrence probabilities of gene attributes. Specifically, our model delves into two pivotal mutation attributes—nucleotide change and mutation type—although its applicability extends to accommodating an array of additional attributes. Given the paramount role of mutations in cellular transformation and the evolution of cancer, they emerge as influential determinants in establishing connections among genes. This, in turn, forms the bedrock of our model's foundation, as it seeks to illuminate the intricate associations that genes foster.

Our approach culminates in the creation of similarity networks, a fundamental representation of genes' mutation data. This innovative framework was not only applied to ovarian cancer mutation data but also extended to encompass a comprehensive case study centered around lung cancer. As a further advancement, we devised local networks, offering a deeper comprehension of inter-gene associations for both driver and non-driver genes within a confined neighborhood. Analyzing both driver and non-driver genes enabled us to explore potential cross-talk and functional connections that might not be immediately evident. This comprehensive approach provided insights into whether non-driver genes, while not primarily linked to cancer initiation, could still play roles in modulating pathways or processes related to the disease's progression.

An integral facet of our research was addressing the intricacies of quantifying categorical data, namely nucleotide change and mutation type. While we addressed these challenges in our current scope, the model's versatility paves the way for future extensions to encompass a broader spectrum of attributes and domains. Our proposed gene similarity measure bears potential for application in unsupervised learning, wherein it can be harnessed to cluster akin genes based on their shared mutation co-occurrences. Beyond the realms of genomics, our model's utility extends to diverse interaction networks encompassing realms such as social networks, collaboration networks, and intricate biological networks encompassing protein–protein interactions, gene/transcriptional regulatory networks, and even the complex chromosomal reengagement network [24].

The current study's limitations stem from its focus on interrelated attributes, assuming that similarity between attribute values is intricately linked to their interactions with other attributes. Yet, there could be numerous other attributes without associations. Another constraint lies in the study's concentration on single point mutations. This disregards the possibility of sequential mutations or causal relationships between them. The method in the manuscript solely examines attribute value co-occurrences and single point mutations, making it unable to directly infer such relationships. Future users of this method should acknowledge these limitations and explore supplementary analyses or approaches to accommodate these intricacies within their datasets.

Crucially, the mathematical framework we have introduced transcends its current domain and can be seamlessly adapted to unearth associations across varied entities within multifaceted networks. The potency of our work lies in its capacity to uncover the latent connections that bind disparate entities, even as these entities sport a tapestry of attributes. In essence, our mathematical model serves as a torchbearer for unveiling the underlying associations interwoven within intricate networks, fostering a deeper understanding of complex systems and phenomena.

## Data Availability

The code underlying this article are available in Github and can be accessed with https://github.com/gm932/gsno. The raw data used in this article can be accessed from COSMIC dataset for Ovarian cancer.

## References

[1] Glazko G, Gordon A, Mushegian A (2005). The choice of optimal distance measure in genome-wide datasets. Bioinformatics.

[2] Barido-Sottan J, Chapman SD, Kosman E (2019). Measuring similarity between gene interaction profiles. BMC Bioinformatics.

[3] Alvarez-Ponce D, Lopez P, Bapteste E, McInerney JO (2013). Gene similarity networks provide tools for understanding eukaryote origins and evolution. Proc Natl Acad Sci U S A..

[4] Wang B, Mezlini A, Demir F (2014). Similarity network fusion for aggregating data types on a genomic scale. Nat Methods.

[5] Navaz AN, El-Kassabi HT, Serhani MA, Oulhaj A, Khalil K (2022). A novel Patient Similarity Network (PSN) framework based on multi-model deep learning for precision medicine. J Pers Med.

[6] Gliozzo J, Mesiti M, Notaro M, Petrini A, Patak A, Puertas-Gallardo A, Paccanaro A, Valentini G, Casiraghi E (2022). Heterogeneous data integration methods for patient similarity networks. Brief Bioinform..

[7] Pai S, Bader GD (2018). Patient similarity networks for precision medicine. J Mol Biol.

[8] Pai S, Hui S, Isserlin R, Shah MA, Kaka H, Bader GD (2019). netDx: interpretable patient classification using integrated patient similarity networks. Mol Syst Biol.

[9] Tian Z, Guo M, Wang C (2017). Constructing an integrated gene similarity network for the identification of disease genes. J Biomed Semant.

[10] Kovács IA, Luck K, Spirohn K (2019). Network-based prediction of protein interactions. Nat Commun.

[11] Bass J, Diallo A, Nelson J (2013). Using networks to measure similarity between genes: association index selection. Nat Methods.

[12] Liu Y, He Q, Sun W (2018). Association analysis using somatic mutations. PLoS Genet.

[13] Bunz F (2008). Principles of cancer genetics.

[14] Chu D, Wei L (2019). Nonsynonymous, synonymous and nonsense mutations in human cancer-related genes undergo stronger purifying selections than expectation. BMC Cancer.

[15] Wei L, Jin Z, Yang S, Xu Y, Zhu Y, Ji Y (2018). TCGA-assembler 2: software pipeline for retrieval and processing of TCGA/CPTAC data. Bioinformatics.

[16] Dhamija S, Yang CM, Seiler J (2020). A pan-cancer analysis reveals nonstop extension mutations causing SMAD4 tumour suppressor degradation. Nat Cell Biol.

[17] Irani J, Pise N, Phatak M (2016). Clustering techniques and the similarity measures used in clustering: a survey. Int J Comput Appl.

[18] Ichino M, Yaguchi H (1994). Generalized Minkowski metrics for mixed feature-type data analysis. IEEE Trans Syst Man Cybern.

[19] Sulc Z, Rezanková H (2019). Comparison of similarity measures for categorical data in hierarchical clustering. J Classif.

[20] Ahmad A, Dey L (2007). A method to compute distance between two categorical values of same attribute in unsupervised learning for categorical data set. Pattern Recogn Lett.

[21] Weinstein JN, Collisson EA, Mills GB, Shaw KR, Ozenberger BA, Ellrott K, Shmulevich I, Sander C, Stuart JM, Cancer Genome Atlas Research Network (2013). The cancer genome atlas pan-cancer analysis project. Nat Genet..

[22] Bastian M, Heymann S, Jacomy M. Gephi: an open source software for exploring and manipulating networks. International AAAI Conference on Weblogs and Social Media. 2009;3(1). 10.1609/icwsm.v3i1.13937.

[23] Lech A, Daneva T, Pashova S, Gagov H, Crayton R, Kukwa W (2013). Ovarian cancer as a genetic disease. Front Biosci.

[24] Mirzaei G (2022). GraphChrom: a novel graph-based framework for cancer classification using chromosomal rearrangement endpoints. Cancers (Basel).

